# Properties of a novel composite elastomeric polymer vinyl polyether siloxane in comparison to its parent materials: a systemic review and meta-analysis

**DOI:** 10.1186/s12903-023-03830-1

**Published:** 2024-01-09

**Authors:** Ravinder S. Saini, Abdulkhaliq Ali F. Alshadidi, Saeed Awod Bin Hassan, Lujain Ibrahim N. Aldosari, Seyed Ali Mosaddad, Artak Heboyan

**Affiliations:** 1 Department of Dental Technology, COAMS, King Khalid University, Abha, Saudi Arabia; 2 Department of Restorative Dental Sciences, College of Dentistry, King Khalid University, Abha, Saudi Arabia; 3 Department of Prosthodontics, College of Dentistry, King Khalid University, Abha, Saudi Arabia; 4 Student Research Committee, School of Dentistry, Shiraz University of Medical Sciences, Shiraz, Iran; 5 Department of Prosthodontics, Faculty of Stomatology, Yerevan State Medical University after Mkhitar Heratsi, Str. Koryun 2, 0025 Yerevan, Armenia

**Keywords:** Vinyl polyether siloxane, Polyether, Polyvinyl siloxane, Elastomeric impression materials, Hybrid impression materials, Fixed prosthodontics

## Abstract

**Background:**

The effectiveness of newly developed elastomeric polymer hybrid siloxane (PVES), which combines the properties of polyethylene (PE) and polyvinyl siloxane (PVS) elastomers, has been a subject of interest in recent studies. This study aimed to assess the physical properties of hybrid PVES materials by analyzing existing data from recent studies on this topic.

**Methods:**

A systematic literature search was conducted to retrieve peer-reviewed articles published up to February 5, 2023. The population, intervention, comparison, and pertinent outcomes were specified under the PICO framework. The primary data analysis was performed in Microsoft Excel, while statistical analysis used Meta-Essentials.

**Results:**

Of the 1152 articles assessed, 14 met the inclusion criteria. The meta-analysis of the selected studies indicated that polyether (PE) and polyvinyl siloxane (PVS) were highly correlated (two-tailed *p*-values of 0.000 and 0.001, respectively) with the improved tensile strength of vinyl polyether siloxane (PVES) with a significantly positive effect size. Similarly, the hydrophilic characteristics of PVES were significantly improved compared to those of PE and PVS. PE was a significant contributor to the hydrophilic characteristics of PVES, with a two-tailed *p*-value of 0.000. The effect size was highly positive for hydrophilicity but showed high heterogeneity. It was also observed that the dimensional accuracy of PVES was comparable to those of PE and PVS, with no statistically significant differences among the three materials.

**Conclusions:**

PVES showed promising features, with improved tensile strength and hydrophilic characteristics compared to those of PE and PVS.

## Background

Ideal impression materials should be flexible, dimensionally stable, hydrophilic, reproducible, have good elastic recovery, have better rheological characteristics, and retain the imitation accuracy of intraoral imprints. In routine clinical dental practice, elastomeric impression materials have far-reaching applicability in the fabrication of dental implants and indirect restorations of the oral cavity [1]. Polysulfides (PSs), polyethers (PEs), polyvinyl siloxanes (PVSs), and silicones are among the most frequently used elastomeric impression materials [2]. These materials have a reputation for their high accuracy and reproducibility. Moreover, contemporary elastomeric materials demonstrate superior dimensional stability and reliable handling characteristics after disinfection, as well as prolonged shelf life. This makes elastomeric impression materials a preferred impression method compared to competing techniques such as digital scanning. Elastomeric impression materials have always been the choice of material in fixed prosthodontics because of their inherent qualities, such as reduced marginal voids and distortion, resulting in improved quality of gypsum dies. In addition to fixed prosthodontics, elastomeric impression materials are used in removable and maxillofacial prosthesis fabrication. These materials accurately record details and aid in the fabrication of well-fitted prostheses [3].

PSs provide excellent surface impressions with accurate reproduction of details as well as sustained dimensional stability. PEs are the oldest elastomeric impression materials with outstanding properties such as hydrophilicity, rigidity, better wettability, and greater elastic recovery. PVSs possess superior dimensional stability and do not release byproducts during the setting period. It also has matchless elasticity, which remains after setting. All elastomers exhibit impressive properties, albeit with some limitations [1]. Silicones are known to produce alcohol as a by-product during the setting process, whereas PSs exhibit suboptimal dimensional stability and a variable half-life. PEs manifest cursory setting times that are expensive and require momentary working time. PVSs, on the other hand, are hydrophobic in nature, which hinders the accuracy of these materials in the presence of moisture and curtails the wettability of tooth surfaces [4]. To address this issue, hydrophilicity is usually attained by adding surfactants to PVSs.

Dimensionally reliable impression materials maintain the accuracy of dental impressions from the time it is applied until the disinsertion of the impression and keep the impression memory unvarying. The hydrophilicity of PEs provides excellent wettability to the materials so that they can make accurate impressions in the presence of saliva on the tooth surface, whereas PVSs maintain 99% elastic memory when the impression is prepared [5]. This prompted the next generation of hybrid impression materials, bolstering the positive attributes of PEs and PVSs, which are termed polyvinyl siloxane ethers or vinyl polyether siloxanes [6]. Vinyl polyether siloxanes are pioneering elastomeric impression materials that can meet the demands of the advanced field of dentistry. This newly created elastomer, vinyl polyether siloxane (PVES), combines the advantages of both polyether and additional silicone. It is a new chemical compound developed by combining a polyether polymer with the vinyl groups of PVS. This new elastomer combines the advantages of polyether and vinyl polysiloxane while also boasting instant hydrophilicity. It combines the hydrophilic properties of polyethers with the impressive elastic memory properties of PVSs [3]. The siloxanes from PVSs account for dimensionally stable impressions with good tear strength; nonetheless, additional traits such as wettability/hydrophilicity and flow properties of the polyether group without adding surfactants [7].

Up-to-scratch mechanical properties facilitate the life impression upon removal from the surface while maintaining dimensional integrity and elastic memory. Notable properties of elastomeric impression materials in dentistry include tensile strength, tear and yield strength, localized deformation, and elastic recovery [1]. PEs exhibit moderate rigidity and higher tensile properties at low viscosities. Tear strength is also pronounced in heavy-body materials [8]. Dimensional stability governs the accuracy of impression materials; elastomeric impression materials show slight contraction upon setting, and PEs are no exception. The higher hydrophilicity of PEs may lead to water absorption when applied to the dental surface. Further shrinkage can occur when water is released during the setting process. PVSs possess higher stress tolerance and better elastic recovery, keeping the impression accuracy intact after deformation when removing the material from the tissues because of their hydrophobic nature [9]. The accuracy of the PVS elastomeric impression materials may fluctuate owing to the rapid polymerization process of the material, which provides a shorter time for cross-linking. However, this makes PVSs ideal for elastic recovery from deformation. The polymerized regions of the PVSs underwent twists and turns, which bore the stress of deformation when the impression was removed, and the material returned to its original state when the stress was removed. It is also pertinent that impression materials break, especially at the margins, when the stress exceeds the recovery limit for making dental undercuts [10, 11]. PVS elastic recovery is also associated with the least permanent deformation, and these materials break rather than undergo permanent deformation when stress is applied beyond the critical point.

Combining PEs with PVSs to create hybrid materials has several mechanical advantages [3]. Because of the comparable mechanical qualities of these two materials, the combination may result in increased tensile and tear strength. Improved performance for dental impressions, where material flexibility and durability are crucial, may come from this synergistic impact. However, there might be difficulties in obtaining the best compatibility and processing conditions, which could impact the mechanical properties [3]. However, a consensus is yet to be reached regarding the impact of combining PE and PVS. This study provides a milestone in the assessment of the mechanical and physical properties of PVES and guidance for the mixing and hybridization of different elastomeric impression materials in dentistry to achieve desirable outcomes.

## Methods

The framework of this systematic review was constructed based on the guidelines set out in the Preferred Reporting Items for Systematic Reviews and Meta-Analyses (PRISMA). The protocol used for this systematic review was the registered International Platform of Registered Systematic Review and Meta-Analysis Protocols (INPLASY) (202330043). The studies were systematically searched in electronic databases. The PICOS strategy included population–elastomeric impression materials, intervention–hybrid elastomeric impression materials (vinyl polyether siloxane), controls–parent impression elastomeric materials (polyvinyl siloxanes and poly ethers), outcome–mechanical properties of hybrid vs. parent elastomers for dentistry application, and source of study – in vitro studies. The Medical Subject Heading (MeSH) term was used to identify studies published before February 5, 2023. The systemic review guidelines were followed according to the instruction of PRISMA with the research question, “Do the hybrid elastomeric impression materials have better elastic memory, tensile strength, hydrophilicity, and dimensional stability than polyether and polyvinyl siloxane?” The sub-quest was, “Does the hybrid vinyl polyether siloxane inherit the positive attributes of poly ethers and polyvinyl siloxanes simultaneously?” The PICO framework was defined as follows to evaluate the physical properties of hybrid PVES materials.

i. Population – Elastomeric impression materials.
ii. Intervention – Hybrid elastomeric impression materials (vinyl polyether siloxane).
iii. Controls – Parent impression elastomeric materials (polyvinyl siloxanes and poly ethers).
iv. Outcome – Physical properties of hybrid vs. parent elastomers for dentistry application.

### Search strategy

A comprehensive literature search of PubMed, Scopus, Embase, and Google Scholar was done up to January 5, 2023. The search was performed to retrieve scholarly articles that comparatively explored the physical properties of PVES, PE, and PVS. The following combinations of phrases were applied: (“vinyl polyether siloxane” OR polyethylene OR “polyvinyl siloxane”) AND (“Mechanical properties” OR “Tensile strength” OR “Tear strength” OR “Hydrophilicity” OR “Dimensional stability” OR “Elastic properties”) AND (dental OR dentistry OR tooth OR teeth OR orofacial OR orthodontics OR prosthodontics). The search phrases were used exhaustively in different combinations in various databases. The combined term search was performed with MeSH terms and the Boolean system. A further hand search was performed to ensure no studies meeting the inclusion criteria were missed and to check the most relevant articles.

### Inclusion and exclusion criteria

The study selection criteria were as follows: 1) in vitro studies, 2) studies should include the standard deviation and mean of mechanical properties of the polyether vinyl siloxanes, 3) studies should have a comparison of PVES with PE and PVS, and 4) publication language. Conference abstract publications, opinions and editorials, patents, and studies with no comparison of properties between PVES vs. PE and PVS were excluded. Clinical trial research studies, theses, and survey reports were excluded. Studies that compared the physical properties of elastomeric impressions after disinfection were excluded. A study was not selected if comparison data were provided as percentages, without sample size and standard deviation of the means of the measurements of the physical properties under investigation. Studies were included without restriction on the publication date. A third researcher independently reviewed the studies to resolve any disagreements.

After removing duplicates, initial screening was performed using titles, abstracts, and full texts, if necessary. Secondary screening involved the selection of studies that compared the mechanical properties of PVES with those of PE and PVS. Finally, the studies were chosen based on data availability, sample sizes, and the type of elastomeric impression materials under consideration.

### Study selection

After removing duplicates, initial screening was performed using titles, abstracts, and full texts—secondary screening involved selecting studies that compared the physical properties of PVES with those of PE and PVS. Finally, the studies were chosen based on data availability, sample sizes, and the type of elastomeric impression materials under consideration.

### Data extraction

Original studies from the corresponding databases were exported using Harzing’s Publish or Perish (Tamra Software Research Ltd) Widows GUI v8.8 edition with MeSH keywords. Data were exported to MS Excel 2021 edition (Microsoft Corporation, Washington, USA) for initial review and assessment by independent reviewers. Initial data extraction included the publication title, abstract, year of publication, authors, publication type, source, and citations. The final sorted data included the type of study, study design and methods, mechanical properties of PE and PVS, mechanical properties of PVES, comparison of the mechanical properties of PVES vs. PE and PVS, publication year, authors, and results.

The properties of elastomeric impression materials for which data were collected for meta-analysis were tensile strength [Pascal (Pa)], elasticity/Young’s modulus [newtons per square meter (N/m^2^)], hydrophilicity/wettability, rigidity [Pascal (Pa)], dimensional stability, viscosity (Newton-second per square meter), and elastic memory. Data were collected for the properties of polyether and polyvinyl siloxanes and their hybrid vinyl polyether siloxanes. Polyether and polyvinyl siloxane were used as reference materials to assess the mechanical properties of the vinyl polyether siloxane. The data collected were mean measurements of mechanical properties, standard deviations, sample size, and *p*-values, where provided.

### Data analysis

Descriptive analysis of data was performed using MS Excel 2021, and meta-analysis was performed using Meta-Essentials 2017 [12]. The mechanical properties of PVES and the parent materials PE and PVS were compared for standard deviation (SD) and standard mean differences. The pooled standard deviation was used to calculate the effect size of the studies as well as the standard error. A two-tailed p-test was performed to determine the correlation of a given mechanical feature of PE or PVS with PVES. A *p*-value < 0.05 was considered statistically significant. The analysis was carried out by comparing the mean values of the mechanical properties of the hybrid PVES elastomeric impression material with those of the parent elastomeric impression materials, PE and PVS. The standard error was used considering the different in vitro techniques used to measure the mechanical properties of the elastomeric impression materials.

The studies were also analyzed for heterogeneity using Cochrane Q and I^2^ values for variance among the results of the selected studies. The studies were analyzed for publication bias. A general trend for the publication of hybrid elastomeric impression materials in dentistry was also assessed with a specific focus on PVES materials. Studies were identified and grouped according to the publication year. Meta-analysis was performed using a random effects model with a 95% confidence interval.

## Results

**Fig. 1:**
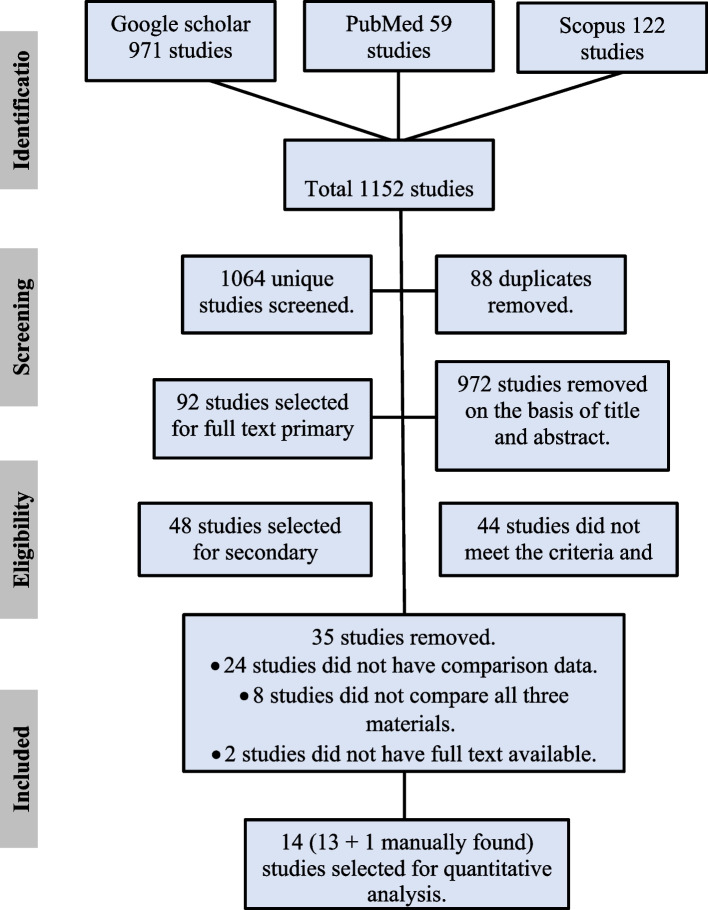
PRISMA flow diagram

The search yielded 1152 potential articles, of which 88 duplicates were removed. Title and abstract screening excluded 972 articles. Ninety-two articles were found to be eligible for full-text analysis. After full-text inspection, 13 studies containing comparison data between hybrid PVES and its parent materials, PE and PVS, were carefully selected. One study was identified through a manual literature search. Finally, 14 studies were included in the meta-analysis. The excluded studies either did not meet the selection criteria or failed to provide sufficient comparison data between PVES, PE, and PVS. The PRISMA flow diagram is shown in Fig. 1.

The properties of the elastomeric impression materials considered for this study were tensile strength or tear strength, hydrophilicity (wettability/contact angle), dimensional accuracy, elastic recovery, detail reproduction, and rigidity. Five studies reported data on tear strength, six reported results on dimensional accuracy, and three reported data on elastic recovery % age of the PVES, PE, and PVS. Four studies reported hydrophilicity data, and two had data available for detailed reproduction. Only one study reported a rigidity comparison between PVES and its parent materials, PE and PVS. A general summary of the studies is shown in Table 1.

**Table 1:** A general summary of the studies selected for meta-analysis

Study	Materials studied	Properties studied	Outcome
Singer^a^ L et al. [13]	PVES, PE, PVS	DA, H, DR	PVES showed improved accuracy and hydrophilicity
Singer^a^ L et al. [14]	PVES, PE, PVS	TS, ER	No significant improvement in PVES
Apinsathanon P et al. [15]	PVES, PE, PVS	TS	PVES has lower tear strength than PVS but higher than PE
Rose S et al. [16]	PVES, PE, PVS	DA, DR	PVES has better accuracy and detail reproduction than PVS and PE
Aivatzidou K et al. [4]	PVES, PE, PVS	DA, DR	No significant differences between PVES, PVS, and PE
Theocharidou A et al. [17]	PVES, PE, PVS	H	No significant difference in hydrophilicity
Elumalai A et al. [8]	PVES, PE, PVS	R	No significant difference in rigidity
Huettig F et al. [18]	PVES, PE, PVS	TS, H	PVES has more tear strength than PE but less than PVS
Pandey P et al. [6]	PVES, PE, PVS	TS, ER	PVS has significantly better performance than PE and PVES in elastic recovery and tensile strength
Emir F et al. [19]	PVES, PE, PVS	DA	PVES has better dimensional accuracy
Mohammed DH et al. [20]	PVES, PE, PVS	DA, H, TS	PVES has lower tear strength than PE but higher than PVS
Re D et al. [2]	PVES, PE, PVS	TS	PVES has the lowest tear strength compared to PE and PVS
Nezam S. et al. [21]	PVES, PE, PVS	DA	PVES has the lowest dimensional accuracy than PE and PVS
Stober T et al. [22]	PVES, PE, PVS	DA	No significant difference in dimensional accuracy

*DA* dimensional accuracy, *H* hydrophilicity, *DR* details reproduction, *TS* tensile strength, *ER* elastic recovery, *R* rigidity

**Fig. 2:**
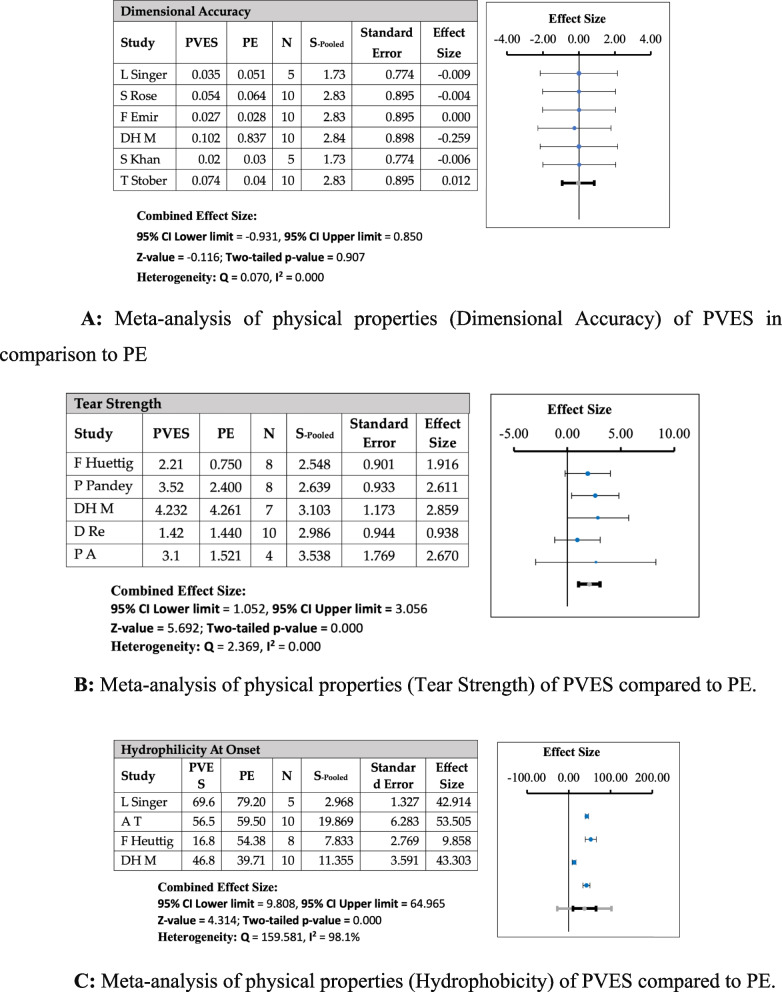
**A** Meta-analysis of physical properties (Dimensional Accuracy) of PVES in comparison to PE. **B** Meta-analysis of physical properties (Tear Strength) of PVES compared to PE. **C** Meta-analysis of physical properties (Hydrophobicity) of PVES compared to PE

**Fig. 3:**
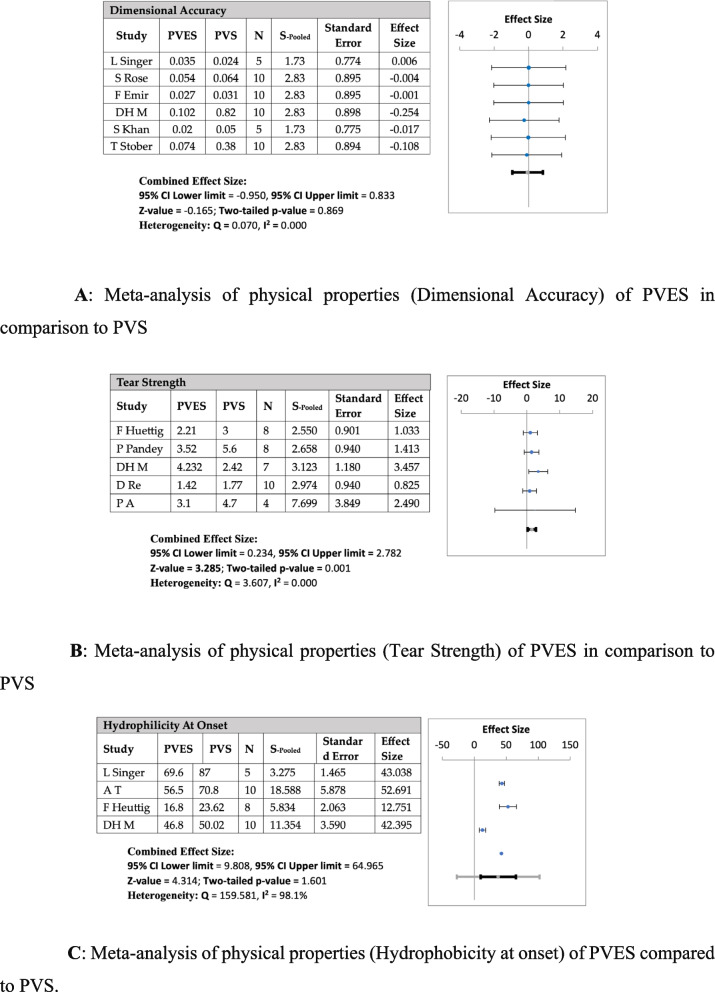
**A** Meta-analysis of physical properties (Dimensional Accuracy) of PVES in comparison to PVS. **B** Meta-analysis of physical properties (Tear Strength) of PVES in comparison to PVS. **C** Meta-analysis of physical properties (Hydrophobicity at onset) of PVES compared to PVS

The meta-analysis showed that PVES inherited tear strength from its parent materials, PE and PVS, showing improved tear strength from both. The results were significant, with a two-tailed *p*-value of 0.000 (0.001 for PVS) and Z-values of 5.69 for PE and 3.285 for PVS, at a 95% confidence interval. The Cochrane Q value for heterogeneity was 2.369, while I^2^ was 0.000, indicating that the results of all studies showed a similar trend. The effect size of the studies showed a significant positive trend. For dimensional accuracy, studies have shown a slightly negative trend toward PVES compared with PVS or PE. However, the overall effect of the hybrid elastomeric impressions on the dimensional accuracy was negligible, with no significant correlation with the polyether and polyvinyl siloxanes (two-tailed *p*-value = 0.907 for PE and 0.869 for PVS). The results are shown in Figs. 2 (A, B, C) and 3 (A, B, C), respectively.

The contact angle measurements of the hydrophilicity of the hybrid impression materials showed a highly positive trend, indicating that the PVS and PE hybrid has improved hydrophilic properties. It was strikingly evident that PE contributed to the highly hydrophilic trait of PVES, as indicated by a two-tailed *p*-value of 0.000 for PE. The combined effect size was 37.39 for all the studies at a 95% confidence interval. Heterogeneity was very high (**I**^**2**^ = 98.12%) for studies that analyzed the contact angle, and a larger sample size is required for a more homogenous outcome.

## Discussion

The purpose of this systematic review was to assess the mechanical properties of hybrid PVES elastomeric impression materials. The PVES hybrid is assumed to inherit the favorable properties of PE and PVS and limit the individual drawbacks of a single material. Several studies have compared the properties of these materials in vitro; however, the results have been inconsistent [19, 20, 22–24]. Recently, there has been growing interest in using PVES in dental impression-making. Achieving the impression of passive fit is crucial for implant success and half-life, which depends on the material’s physical properties, including tensile strength, dimensional accuracy, and hydrophilicity.

PVES inherited some of the positive attributes of both PE and PVS. Superior PVES tear strength was consistently reported among the studies, and statistical analysis validated the results. Tear strength significantly contributes to stress absorption during impression removal. It is also a determining factor for the impression material to maintain the impression replica of thin areas of a tooth, such as crevices and proximal regions of the impression. It has been established that PVS has better tear strength than PE, and hybridizing these two materials into single elastomeric materials results in a far superior tensile property than these parent materials, as proposed in the null hypothesis of this review. The included studies’ homogeneity (I^2^ = 0.000) indicated that the data were highly consistent. The highest tear strength measurements of PVES were reported by Mohammad et al. (4.232 MPa) [20], while the lowest measurement was 1.42 MPa, observed by Re et al. [2]. All studies reported higher PVS tensile strength against PE except for one study.

Tearing of impressions is initiated under high stress and is commonly caused by defects in material consistency, poor elasticity, and deformation during the recovery of impressions. The improvement in tensile strength in the polyether and polyvinyl hybrid indicates that these materials reinforce each other during polymerization, maintaining individual elasticities but being resistant to deformation. Another factor contributing to the tensile properties of elastomeric impression materials is their ability to polymerize in a given time, which is primarily governed by the chemical composition. A poorly polymerized or incompletely polymerized material does not bear the stresses of removal and tearing [25]. Improved tear strength suggests that the polymerization ability of PE and PVS in the mixture was better. Singer et al. reported that the PVES hybrid showed lower tear strength than its parent materials, PE and PVS, but the results were statistically insignificant. It was also observed that the ratio of tear strength at break (TB) to tensile strength (TS) is a more predictive factor for clinically significant applicability than the tensile strength of the impression material alone. A TB/TS ratio of 1 is considered ideal, and the PVES is 0.8, higher than PE or PVS elastomers.

The statistical analysis of dimensional accuracy data from the studies showed that, in general, there was no significant difference in the dimensional accuracy of hybrid PVES compared to PE or PVS. According to the American Dental Council, tan impression material is considered accurate if it can replicate details up to 25 μm [14]. All elastomeric impression materials can measure details up to 1 – 2 μm. PVS materials are generally preferred over PE materials to make more accurate impression materials. However, in this study, there was no significant difference (two-tailed *p*-value > 0.05) between the impression accuracies of all three elastomeric impression materials. This indicates that the dimensional accuracy of PVES is not significantly better than that of PVS or PE. These results also support Stober et al. [22] and Aivatzidou et al. [3], who reported insignificant differences in dimensionally accurate impressions produced by PVES, PE, and PVS. Among the selected studies, Singer et al. [13] and Rose et al. [16] reported significantly improved dimensional accuracy with PVES compared to PE and PVS. Further research is required in this regard.

The dimensional accuracy measurements involved different methods, including direct measurements and comparison of impressions with a master mold made of metal, plaster, or acrylic. Another factor that could explain this variability is humidity. The water content significantly hinders accuracy, which is why PVS possesses better impression accuracy owing to its inherent hydrophobic characteristics. Furthermore, it has been reported that impression accuracy is influenced by temperature. This requires careful consideration of the physical parameters during impression-making to more precisely assess the dimensional stability of PVES, PE, and PVS.

The hydrophilic or wettable characteristics of impression materials also contribute to passively fitting impressions. The hydrophilicity was measured by determining the contact angle of a drop of water on the material under observation [17]. The lower the hydrophilicity, the larger the angle, and the higher the discrepancy in the impression. The selected studies were highly heterogeneous. PE was the main contributor to the hydrophilic properties of PVES, with a two-tailed *p*-value of 0.000, whereas for PVS, a two-tailed test showed no significant correlation (*p* = 1.60) owing to its hydrophobic properties. PVS generally requires the addition of surfactants or hydrophilic silicones to obtain the desired hydrophilicity. In the PVES hybrid, the hydrophilicity of PE is sufficient for PVS to become hydrophilic. Hydrophobic materials such as PVS show a contact angle greater than 90° with water. The data obtained from the selected studies showed a general trend of higher contact angles for PVS than PE and PVES. Surprisingly, Heuttig et al. [18] reported the lowest contact angle of 23.62° for PVS, which was even lower than that of PE (54.38°) but slightly higher than that of PVES (16.8°). The study did not mention a specific reason for such a lower contact angle. A possible explanation for this reduced contact angle could be the impact of processing parameters during PVS material production. Certain processing variables, such as temperature and curing techniques, can significantly affect the material’s surface properties.

Singer et al. reported that PVES demonstrates improved hydrophilicity and wettability compared to PE and PVS, and these observations were also consistent with other studies, except Mohammad et al., who reported less PVES wettability compared to PE. Heuttig et al. reported the highest hydrophilicity of PVES [18], with a mean contact angle of 16.8° among eight samples. Precise and accurate impressions are due to good flow and wetting of the tooth surface with elastomeric impression materials, where hydrophilicity plays a crucial role in the final set, and the impression result [26]. Precision may also be influenced by saliva, which has a distinct composition of salt, proteins, and mucin. In clinical settings, the impression material interacts with saliva, and its components may play a role in the hydrophilic wettability of the material when interacting with adhesive mucins. A reliable and more accurate way to assess the hydrophilic property of an elastomeric impression material should be performed using a drop of saliva instead of water and then measuring the contact angle.

The data for elastic recovery, detailed reproduction, viscosity (light-body, medium-body, and heavy-body materials), and rigidity were insufficient to provide conclusive results when comparing PVES, PE, and PVS properties. Generally, PVS provides slightly better elastic recovery results than PE. Pandey et al. [6] reported that the elastic recovery of PVES was comparable to that of PVS and PE with no significant difference, and the same results were reproduced by Singer et al. and Heuttig et al. [18]. As elastic recovery is mainly linked to the polymerization of a given elastomer with monomers storing the potential energy of restoration during deformation, a more detailed analysis of elastic recovery may also need to consider the chemical properties for polymerization.

The detailed reproduction of PVES is comparable to that of PE and PVS, as reported by Singer et al. Aivatzidou et al. [4] reported that the reproductive ability of PVES is similar to that of PVS but less than that of PE, which is generally well-known for its superior impression details. In contrast, Rose et al. [16] observed that PVES and PE showed similar detailed reproductive traits and that PVS was the worst among the three. However, further research is required to make a conclusive remark on the elastic recovery of PVES and its detailed reproduction. Regarding the rigidity of PVES, Elumalai et al. [8] reported that PVES was the least rigid compared with PE and PVS, and the difference was significant (*p* = 0.001). Rigidity is often associated with elastic modulus. This governs the elastic memory of the impression when removed from the oral cavity [24]. Removal requires the application of stress, which can be a driving force for defects and deformations in impressions. More data are necessary to analyze the rigidity of hybrid elastomeric impression materials compared to PE and PVS.

In a clinical setting, elastomeric impression materials undergo different disinfection procedures. The chemical or physical techniques used to disinfect these impression materials may also contribute to the discrepancies induced by the interaction of the polymer with the chemicals [27–29]. The clinical outcomes of impressions designed from these elastomers must also be compared to obtain a clearer understanding of the material that would be more relevant and reliable under clinical conditions. Another aspect worth considering during comparative studies of elastomeric impression materials is the controlled temperature and humidity, which can mimic clinical settings.

## Conclusions

The results of this systematic review provide a clearer understanding of the properties of novel PVES hybrid impression materials. PVES has significantly better tensile strength and provides impressions with fewer defects compared to PE and PVS. PVES exhibited better wettability and contact angle with water than PVS and PE. This makes it an excellent alternative to PVS, in which moist conditions are prevalent in the clinical setting. However, the dimensional stability of PVES is comparable to that of PE and PVS. This makes PVES the preferred material for dental impressions. It can be concluded that PVES hybrid elastomers eliminate the drawbacks of PE and PVS, providing a novel impression material with superior tensile strength, hydrophilicity, and dimensional accuracy in one place. It is also worth noting that PVES exhibits satisfactory elastic recovery and detailed reproduction, as individual studies indicate. However, further research is imperative for a more detailed analysis of elastic recovery and reproduction. If elastic recovery is a priority for making dentures or diagnostic purposes, PE may be the preferred choice to avoid uncertainty. The same is true for PVS, which can imprint finer details during impression-making and could be a desirable choice where necessary. Owing to the limited data available for a complete assessment of all the physical properties of the PVES in comparison to PE and PVS, more research is required.

## Data Availability

The data supporting this study’s findings are available from the corresponding author upon reasonable request.

## Abbreviations

PS: Polysulfide
PE: Polyether
PVS: Polyvinyl siloxane
PVES: Vinyl polyether siloxane
PRISMA: Preferred Reporting Items for Systematic Reviews and Meta-Analyses
INPLASY: International Platform of Registered Systematic Review and Meta-Analysis Protocols
MeSH: Medical Subject Heading
Pa: Pascal
N/m2: Newtons per square meter
SD: Standard deviation
DA: Dimensional accuracy
H: Hydrophilicity
DR: Details reproduction
TS: Tensile strength
ER: Elastic recovery
R: Rigidity
TB: Tear strength at break
